# Der Stellenwert der Eingliederungshilfe bei der Versorgung schwer psychisch Kranker

**DOI:** 10.1007/s00115-025-01897-5

**Published:** 2025-09-19

**Authors:** Raoul Borbé, Iris Graef-Calliess, Gerhard Längle

**Affiliations:** 1 Klinik I für Psychiatrie und Psychotherapie der Universität Ulm, Weingartshoferstr. 2, 88214 Ravensburg, Deutschland; 2 ZfP Südwürttemberg, Ravensburg-Weissenau, Deutschland; 3 Universitätsklinik für Psychiatrie und Psychotherapie, Tübingen, Deutschland; 4 ZfP Südwürttemberg, Zwiefalten, Deutschland

**Keywords:** Assistenzleistung, Teilhabe, Sozialgesetzbuch (SGB) IX, Personenzentrierung, Kooperation, Assistance service, Participation, Social Security Code IX (SGB IX), Person centeredness, Cooperation

## Abstract

**Hintergrund:**

Menschen mit schweren psychischen Störungen brauchen häufig auch zwischen akuten Krankheitsphasen multiprofessionelle Behandlung und Unterstützung. Dabei wechseln sie immer wieder zwischen stationärer Akutversorgung und ambulanter gemeindepsychiatrischer Betreuung im Rahmen der Eingliederungshilfe.

**Fragestellung:**

Was ist Eingliederungshilfe? Welche Aufgaben bei der Versorgung schwer psychisch kranker Menschen werden durch die Eingliederungshilfe erfüllt? Wie kann die Zusammenarbeit zwischen Akutpsychiatrie und Eingliederungshilfe gelingen?

**Material und Methode:**

Selektive Literaturübersicht.

**Ergebnisse:**

Die Eingliederungshilfe nach Sozialgesetzbuch (SGB) IX dient der Unterstützung bei gesellschaftlicher Teilhabe. Dieser Bedarf ist bei der Gruppe der schwer psychisch Kranken häufig gegeben. Assistenzleistungen der Eingliederungshilfe sind flexibel und bedürfnisorientiert einsetzbar. Die sektorenübergreifende personenzentrierte Behandlung dieser Patientengruppe bedarf einer engen Abstimmung zwischen Kliniken, ambulanten Behandlern und Eingliederungshilfe, bspw. durch Fallkonferenzen und Case-Management.

**Schlussfolgerungen:**

Kliniken, ambulante Behandler und Leistungserbringer der Eingliederungshilfe sollten bei schwer psychisch Kranken eng miteinander kooperieren, um soziale Teilhabe zu ermöglichen.

## Abstract (en)

**Background:**

People with severe mental disorders often need multiprofessional treatment and support, even between acute phases of illness. During the integration support they repeatedly switch between inpatient acute care and outpatient community psychiatric care.

**Objective:**

What is integration support? What tasks in the care of severely mentally ill people are fulfilled by integration assistance? How can cooperation between acute psychiatry and integration support be successful?

**Material and method:**

Selective literature search.

**Results:**

The integration support according to the Social Security Code IX (SGB IX) serves to support social participation. This need is often present in the group of severely mentally ill people. Integration support services can be used flexibly and needs oriented. The cross-sectoral, person-centered treatment of this patient group requires close coordination between clinics, outpatient therapists and integration support, for example by case conferences and case management.

**Conclusion:**

Clinics, outpatient practitioners and providers of integration support should closely cooperate with each other to provide social inclusion for severe mentally ill patients.

Eine individuelle Versorgungsplanung ist entscheidend für eine langfristige Stabilisierung und Verbesserung der Lebensqualität bei Menschen mit schweren psychischen Störungen. Für den Ausgleich krankheitsbedingter Teilhabedefizite besteht ein Rechtsanspruch auf Leistungen der Eingliederungshilfe. Die Verbesserung der sozialen Teilhabe muss schon in der Klinik geplant werden.

## Hintergrund

Auch bei suffizienter Behandlung der Akutsymptomatik leiden viele Menschen mit schweren psychischen Störungen an anhaltenden kognitiven Einschränkungen, Motivations- und Antriebsstörungen sowie dysfunktionalen Einstellungen [20]. Dies kann die gesellschaftliche Teilhabe teils erheblich, langfristig und bereits in jüngerem Alter beeinträchtigen, mit der Folge sozialer Exklusion [19]. Gezielte Interventionen haben einen positiven Einfluss auf den Selbstwert der Betroffenen, die Adhärenz zu anderen Therapiemaßnahmen, aber vor allem auf das Ziel der gesellschaftlichen Inklusion, das auch explizit eines der psychiatrisch-psychotherapeutischen Versorgung ist [14].

In der Bundesrepublik Deutschland übernimmt diese Aufgabe die Eingliederungshilfe (EGH), die nach Sozialgesetzbuch (SGB) IX § 90 die Ermöglichung einer individuellen Lebensführung *und* die Förderung der gleichberechtigten Teilhabe am Leben in der Gesellschaft realisieren soll [5].

Ziel dieses Artikels ist, die historische Entwicklung der Eingliederungshilfe kurz zu skizzieren, deren neue gesetzliche Grundlagen und ihren Auftrag darzustellen, die Bedeutung bei der Versorgung schwer psychisch kranker Menschen zu illustrieren und die Schnittstelle zur klinischen Psychiatrie zu beleuchten.

## Material und Methoden

Eine selektive Literaturrecherche wurde in PubMed und Google durchgeführt, wobei die Suchbegriffe „Eingliederungshilfe“, „Kooperation“, „Personenzentrierung“, „Teilhabe“, „integration support“, „severe mental ill“, „participation“ „person centredness“ „cooperation“ und „psychiatry“ verwendet wurden. Die in dieser selektiven Literaturübersicht („narratives Review“) enthaltenen Artikel wurden nicht auf systematischer Basis ausgewählt, und es wird nicht davon ausgegangen, dass die rezensierte Evidenz erschöpft ist. Die identifizierten Artikel und Buchkapitel wurden anschließend im Konsens zwischen den Autor:innen diskutiert.

## Historische Entwicklung der Eingliederungshilfe

Die Eingliederungshilfe wurde 1962 im Bundessozialhilfegesetz festgeschrieben und fokussierte zunächst auf Menschen mit geistiger Behinderung [2]. Erst mit der Novelle 1974 wurde ein einheitlicher Behindertenbegriff geschaffen, was zeitlich einherging mit der beginnenden Reform des psychiatrisch-psychotherapeutischen Versorgungssystems. Deren Auslöser war vor 50 Jahren der „Bericht über die Lage der Psychiatrie in der Bundesrepublik Deutschland“ der Enquetekommission des Deutschen Bundestages [9]. Bis in die 1970er-Jahre lebten viele psychisch Kranke, insbesondere die mit chronischen Erkrankungen, dauerhaft in Kliniken, da diese neben der psychiatrischen Akutversorgung auch häufig für die Langzeitbetreuung, Begleitung und Pflege zuständig waren [7].

Bei der Entwicklung ambulanter und stationärer Betreuungs- und Versorgungsangebote übernahmen vielfach Einrichtungen der freien Wohlfahrtspflege die Initiative. Insbesondere Konzepte zur Unterstützung beim Wohnen in der Alltagsgestaltung, der Tagesstruktur und der leistungsangepassten Arbeitsmöglichkeiten wurden entwickelt. Ein Großteil dieser Leistungen gehört heute zum Leistungsangebot der Eingliederungshilfe.

Die Entwicklung des personenzentrierten Ansatzes in der psychiatrisch-psychotherapeutischen Versorgung, federführend entwickelt durch die Aktion Psychisch Kranke e. V. in den 1990-er Jahren, wies einer individualisierten, bedürfnisorientierten Versorgungsplanung den Weg. Die Ratifizierung der UN-Behindertenkonvention (UN-BRK) durch die Bundesrepublik Deutschland im Jahre 2009 leistete dieser einen weiteren Vorschub mit dem Ziel der „vollen, wirksamen und gleichberechtigten Teilhabe an der Gesellschaft“ [5]. Daher wurde auch eine Reform der Eingliederungshilfe notwendig.

## Reform der Eingliederungshilfe durch das Bundesteilhabegesetz

Durch das Bundesteilhabegesetz (BTHG) wurde die Eingliederungshilfe aus dem SGB XII in das SGB IX überführt und damit der Schwerpunkt weg von der sozialen Sicherung hin zu Rehabilitation und Teilhabe verlagert [6]. Durch Betonung des Wunsch- und Wahlrechts soll die gleichberechtigte Partizipation und Inklusion Behinderter gefördert werden. Das SGB IX sieht dafür fünf Leistungsgruppen zur Teilhabe an der Gesellschaft vor:

1. Leistungen zur medizinischen Rehabilitation
2. Leistungen zum Arbeitsleben
3. Unterhaltssichernde und andere ergänzende Leistungen
4. Leistungen zur Teilhabe an Bildung
5. Leistungen zur sozialen Teilhabe

Die Intensität der Betreuung und Unterstützung soll nicht mehr abhängig sein von der Wohnform. Die Komplexversorgung in Fachpflegeheimen und anderen stationären Wohnangeboten, die in vielem noch an den alten Anstaltsstrukturen orientiert waren, sollen ab- und aufgelöst werden.

Hier entfaltet das BTHG bereits Steuerungswirkung. So nimmt seit einigen Jahren die Zahl der Leistungsempfänger in besonderen, d. h. stationären Wohnformen ab, während die Zahl derer, die Assistenzleistungen erhalten (was in etwa dem ambulant betreuten Wohnen entspricht), immer weiter zunimmt [1].

## Eingliederungshilfe für Menschen mit schweren psychischen Störungen

Professionell Tätige, ob Ärzt:innen, Psycholog:innen oder Mitarbeitende aus anderen Berufsgruppen, sollten die basale Nomenklatur und die Eingangsvoraussetzungen der Eingliederungshilfe kennen, wenn sie Menschen mit schweren psychischen Störungen behandeln (Tab. 1).

**Tab. 1:** Definitionen

Die Eingliederungshilfe ist eine Leistung des SGB IX – Rehabilitation und Teilhabe für Menschen mit Behinderungen (und von Behinderung bedrohte Menschen)
Träger der Eingliederungshilfe oder auch Kostenträger sind die Land- und Stadtkreise oder Landschaftsverbände
Leistungserbringer im SGB IX sind Erbringer sozialer Dienstleistungen, weitestgehend Organisationen in freigemeinnütziger Trägerschaft, aber auch Kliniken
Die Wiedereingliederung ist eine Leistung des SGB V (§ 74) und dient der stufenweisen Wiedereingliederung in das Erwerbsleben
Eine *Wiedereingliederungshilfe* gibt es nicht, wenngleich dieses Wort oft im fachlichen Diskurs fälschlicherweise für die Eingliederungshilfe verwendet wird

*SGB* Sozialgesetzbuch

Die S3-Leitlinie Psychosoziale Therapien bei schweren psychischen Erkrankungen geht von einer Prävalenz von 1–2 % der Bevölkerung mit einer schweren psychischen Erkrankung aus [13]. Damit wären 0,8–1,6 Mio. Menschen betroffen. Aktuelle Zahlen der Kostenträger zeigen, dass in Deutschland etwa 232.000 Menschen mit seelischer Behinderung Leistungen der Eingliederungshilfe erhalten [1]. Auch wenn man nur von einer Prävalenz von 1 % ausgehen würde, läge die Quote der durch EGH versorgten Menschen mit schwerer psychischer Erkrankung nur bei knapp 30 %. Die Gründe dafür sind vielfältig und können hier nicht im Einzelnen ausgeführt werden. Es zeigt aber, dass für Patient:innen, die bisher keine Leistungen der Eingliederungshilfe erhalten, diese im Rahmen einer klinischen Krisenintervention immer in Erwägung gezogen werden sollten, selbst wenn Patient:innen zunächst ablehnend sind oder Eingliederungshilfeleistungen vermeintlich nicht erbracht werden können, bspw. bei Wohnungslosigkeit.

Die wichtigsten Kriterien für den Erhalt von Leistungen der Eingliederungshilfe sind:

- Vorliegen einer wesentlichen Behinderung, die länger als sechs Monate besteht oder voraussichtlich bestehen wird.
- Die Teilhabe am Leben in der Gesellschaft ist wesentlich beeinträchtigt.
- Es liegt ein individueller Bedarf an Unterstützung vor.

Liegen nun aus fachpsychiatrischer Sicht bei einem Betroffenen eine schwere psychische Störung und Teilhabedefizite vor, so kann ein Antrag auf Leistungen der EGH beim zuständigen Kostenträger durch den Betroffenen gestellt werden. Durch einen Facharzt muss das Vorliegen einer wesentlichen Behinderung attestiert werden.

Die eigentliche Teilhabeplanung oder Gesamtplanung wird durch das Fallmanagement des Leistungsträgers zusammen mit dem Betroffenen vorgenommen. Diese Änderung durch das BTHG ist hoch umstritten, da nun der Kostenträger nach Anhörung des Leistungsberechtigten die Versorgungsplanung macht und letztverantwortlich über die Leistungsgewährung entscheidet. Allerdings kann der Betroffene eine Person seines Vertrauens hinzuziehen, bspw. den Kliniksozialarbeiter, dem im klinischen Setting die Schlüsselrolle in der Umsetzung des BTHG zufällt [11].

## ICF-basierte Teilhabeplanung stärkt den personenzentrierten Ansatz

Im § 118 SGB IX wurde festgelegt, dass „die Ermittlung des individuellen Bedarfs des Leistungsberechtigten […] durch ein Instrument erfolgen [muss], das sich an der Internationalen Klassifikation der Funktionsfähigkeit, Behinderung und Gesundheit orientiert“ [6]. Die ICF (International Classification of Functioning, Disability and Health) gehört zur WHO-Familie der internationalen Klassifikationen (World Health Organization, WHO) und wurde 2001 veröffentlicht [10].

**Abb. 1:**
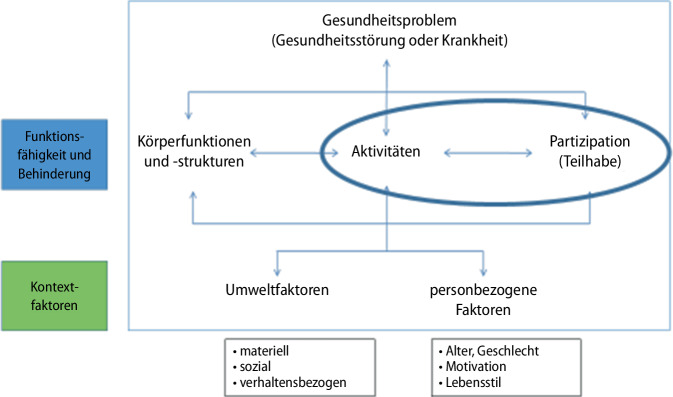
ICF (International Classification of Functioning, Disability and Health) in der Teilhabeplanung. (Mod. nach [10])

Die Grundsätze der ICF entsprechen in vielem dem biopsychosozialen Denkmodell der Psychiatrie und sind insofern auf die Betrachtung und Behandlung psychischer Krankheiten sehr gut anzuwenden (Abb. 1). Mit der ICF wird der Blick nicht, wie im medizinischen Kontext oft üblich, auf die Einschränkungen und Beeinträchtigungen gerichtet, sondern gleichwertig auf die Ressourcen, Kompetenzen und die bereits vorhandenen sozialen Unterstützungsprozesse. Entscheidender Endpunkt aller Bemühungen und die Bewertung des Behandlungs- und Betreuungserfolges ist die soziale Teilhabe.

Die Entwicklung der ICF-basierten Bedarfsermittlungsinstrumente (BEI), die der Teilhabeplanung und der Bedarfsfeststellung zugrunde liegen, liegt allerdings in Länderhoheit. Der baden-württembergische BEI-BW bspw. wurde von 41 auf 8 Seiten gekürzt und ist alleine durch das Fallmanagement des Kostenträgers auszufüllen. Das gilt auch für den medizinischen Teil, der auf der Basis von Arztbriefen und anderen Unterlagen erstellt wird. Dies wird von verschiedenen Seiten kritisch gesehen, zeigt aber auch die Notwendigkeit, Aspekte der Teilhabe im Arztbrief konsequent zu berücksichtigen.

## Assistenzleistungen

Assistenzleistungen gehören zu den Leistungen für soziale Teilhabe und können als Kern der Flexibilisierung der Eingliederungshilfeleistungen durch das BTHG gesehen werden [16]. Vor der Reform war die Unterstützung meist an den Wohnraum gekoppelt, die Versorgungsplanung wurde wenig differenziert formuliert und die Hilfen oft als Leistungspaket und nicht individualisiert erbracht. Um das umfassende Ziel der „selbstbestimmten und eigenständigen Bewältigung des Alltags“ zu erreichen, werden in § 78 SGB IX Abs. 1 insbesondere die Lebensbereiche aufgezählt, in denen die Betroffenen durch Assistenzleistungen unterstützt werden sollen. Dazu gehören Haushaltsführung, soziale Beziehungen, persönliche Lebensplanung, gemeinschaftliches und kulturelles Leben und Freizeitgestaltung. Besonders hinzuweisen ist auch auf die Sicherstellung der Wirksamkeit der ärztlichen und ärztlich verordneten Leistungen und die Leistungen an Mütter und Väter mit Behinderungen bei der Versorgung und Betreuung ihrer Kinder. Detaillierter führen das die jeweiligen Landesrahmenverträge aus (Tab. 2).

**Tab. 2:** Ziele der Unterstützung durch Assistenzleistungen. (Mod. nach [16])

Sicherstellung und Erledigung alltäglicher Aufgaben und Routinen
Sicherstellung der Selbstversorgung
Vermittlung eines positiven Umgangs mit der Behinderung
Bewältigung der Gesundheitssorge im alltagspraktischen Kontext
Strukturierung des Alltags
Unterstützung und Förderung der Spiritualität, von Hobbys
Förderung des gesellschaftlichen und politischen Engagements
Unterstützung und Förderung bei der Teilnahme an sportlichen, kulturellen, gesellschaftlichen und politischen Veranstaltungen
Aufbau und Aufrechterhaltung sozialer Beziehungen, Vermeidung von Isolation
Auseinandersetzung mit der eigenen Lebenssituation und Entwicklung und Umsetzung von Zukunftsperspektiven und Interessen in den Bereichen Bildung, Ausbildung, Arbeit, Wohnen, Partnerschaft, Familienplanung und soziale Sicherheit
Erhaltung und Verbesserung des Gesundheitszustandes unter Anwendung biopsychosozialer Modelle
Umgang mit Belastungssituationen und Stärkung der Resilienz durch Inanspruchnahme medizinischer Versorgung und Umsetzung ärztlicher und therapeutischer Anordnungen und Empfehlungen

Für den klinischen Alltag bedeutet dies, dass bei Aufnahmen schwer erkrankter Patient:innen in klinische Settings die Sozialanamnese diese Bereiche miterfassen muss.

Die Erfassung findet idealerweise immer unter Beteiligung der Betroffenen statt, ist aber aufwendig und wird erleichtert durch die Verbesserung der Behandlungskontinuität, der Vernetzung der Versorgung und einen kontinuierlichen Informationsfluss. Das Track-System ist ein Ansatz, in dem diese Kriterien weitestgehend erfüllt werden und bedürfnisorientierte Versorgung auch im stationären Setting schnell realisiert werden kann [8]. Die Patient:innen werden bei Krisen immer den gleichen Behandlungseinheiten zugewiesen und haben die Möglichkeiten, innerhalb dieser Behandlungseinheit in einem Setting behandelt zu werden, das der notwendigen Betreuungsintensität gerecht wird. Dies erleichtert auch eine sektorenübergreifende, überdauernde Beziehungsarbeit.

## Zum Verhältnis von klinischer Psychiatrie und Eingliederungshilfe

Eingliederungshilfe und Kliniken bilden eine wichtige Schnittstelle bei der Versorgung von Menschen mit schweren psychischen Störungen. In beiden Settings zeigt sich bei dieser Subgruppe häufiger herausforderndes Verhalten, mangelnde therapeutische Adhärenz und konsekutiv vermehrt Krisen. Dabei muss man sich immer vergegenwärtigen, dass die EGH viele Krisen häufig bereits im Lebensumfeld der Betroffenen abfängt, ggf. unterstützt von ambulanten Therapeuten und Krisendiensten [12].

Der Blickwinkel der in der Klink Tätigen ist oft verkürzt und konkret begrenzt auf die Zeit der stationären, der tagesklinischen oder stationsäquivalenten Behandlung. Zugespitzt formuliert fokussiert die Klinik auf Symptomfreiheit, die Eingliederungshilfe auf Teilhabe, wobei der Alltag vieler Menschen mit schwerer psychischer Störung durch den Wunsch nach Teilhabe *trotz* Residualsymptomatik geprägt ist. Um dies bestmöglich zu realisieren, ist eine enge Zusammenarbeit aller Beteiligten notwendig.

Die im klinischen Kontext weitestgehend übliche Pflichtversorgung für eine Versorgungsregion ist im Bereich der außerklinischen Versorgung nach SGB IX allerdings bisher nicht vorgesehen. Für die Umsetzung einer funktionalen Versorgungsmatrix aus klinisch-psychiatrischer (SGB V) und teilhabeorientierter gemeindepsychiatrischer Versorgung durch die Eingliederungshilfe (SGB IX) wäre diese Anpassung der Verbindlichkeit aber eine wichtige Voraussetzung [3]. Insofern bleibt die Versorgung der schwer- und schwerstkranken Personen und der Personen mit herausforderndem Verhalten eine freiwillige Leistung der jeweiligen Anbieter von Unterstützung nach SGB IX.

Die daraus resultierenden Problemlagen für die Pflichtversorgung der Krankenhäuser sind vielfach benannt, im Besonderen die Zunahme an Langliegern [15], weswegen eine aktive Rolle der psychiatrischen Kliniken in gemeindepsychiatrischen Verbundstrukturen umso wichtiger ist [4].

Neben einer frühen Versorgungsplanung in der Klinik unter Einschluss der Eingliederungshilfe, was als „good clinical practice“ betrachtet werden sollte, gibt es noch spezifische Ansätze, die eine personenzentrierte Behandlung unterstützen.

Das Track-System, das eine hohe Behandlungskontinuität über Grenzen der Behandlungssektoren hinweg sichert, wurde oben schon genannt. Zum anderen kann die klinische Versorgungsplanung für den Übergang in den ambulanten Bereich durch ein intensiviertes, personenzentriertes Fallmanagement verbessert werden [18]. Neue innovative Modelle, wie bspw. globale Behandlungs- und Teilhabebudgets, fokussieren darauf, nach Möglichkeit rechtskreisübergreifend jegliche Schnittstellen in einem Gesamtbehandlungsplan zu minimieren bzw. abzuschaffen [21]. Dies könnte auch die Steuerung zwischen Akutversorgung durch die Klinik und Teilhaberealisierung durch Unterstützung der Eingliederungshilfe deutlich erleichtern.

## Schlussfolgerung

Eingliederungshilfe ist nach der Reform durch das BTHG eine Leistung des SGB IX, mit Betonung von Rehabilitation und Inklusion, weg von Fürsorge und Versorgung. Die Eingliederungshilfe sichert bei vielen Menschen mit schweren psychischen Erkrankungen die gesellschaftliche Teilhabe alltagsnah in ihrem Lebensumfeld. Für die akute Krisenintervention und intensivierte Behandlung ist sie auf die Kliniken angewiesen. Betroffene profitieren daher von einer gemeinsamen Versorgungsplanung durch Kliniken und Eingliederungshilfe in gemeindepsychiatrischen Verbundstrukturen.

## Fazit für die Praxis

- Die Eingliederungshilfe (EGH) sichert über Assistenzleistungen gesellschaftliche Teilhabe.
- Krisen können in der EGH früh erkannt werden. Durch enge Kooperation mit ambulanten Behandlern können Klinikeinweisungen reduziert werden.
- Pflichtversorgung in der Eingliederungshilfe würde den Grad der Verbindlichkeit in solchen Versorgungsstrukturen erhöhen.
- Innovative Versorgungs- und Finanzierungsmodelle sind notwendig, um eine sektorenübergreifende Steuerung zu ermöglichen.
